# Differential effect of covalent protein modification and glutathione depletion on the transcriptional response of Nrf2 and NF-κB^☆^

**DOI:** 10.1016/j.bcp.2010.04.004

**Published:** 2010-08-01

**Authors:** Alvin J.L. Chia, Christopher E. Goldring, Neil R. Kitteringham, Shi Quan Wong, Paul Morgan, B. Kevin Park

**Affiliations:** a MRC Centre for Drug Safety Science, Department of Pharmacology and Therapeutics, University of Liverpool, Liverpool, Merseyside, L69 3GE, UK; b Pfizer Global Research and Development, Department of Pharmacokinetics, Dynamics and Metabolism, Sandwich, Kent, CT13 9NJ, UK

**Keywords:** DILI, drug-induced liver injury, CRMs, chemically reactive metabolites, GSH, glutathione, Nrf2, nuclear factor-erythroid 2 (NF-E2)-related factor, NF-κB, NF-kappa B, ARE, anti-oxidant response element, NAPQI, N-acetyl-p-benzoquinineimine, DNCB, dinitrochlorobenzene, BSO, buthionine (S,R)-sulfoximine, RNAi, RNA interference, Nrf2, Keap1, NF-κB, RNAi, Cellular stress, Transcriptional response

## Abstract

Liver injury associated with exposure to therapeutic agents that undergo hepatic metabolism can involve the formation of reactive metabolites. These may cause redox perturbation which can result in oxidative stress as well as protein modification leading to activation or inhibition of cellular transcriptional responses. Nevertheless, the effects of these challenges on more than one transcriptional pathway simultaneously remain unclear. We have investigated two transcription factors known to be sensitive to electrophilic stress and redox perturbation, Nrf2 and NF-κB, in mouse liver cells. Cellular stress was induced by the probes: N-acetyl-p-benzoquinineimine (NAPQI), the reactive metabolite of acetaminophen; dinitrochlorobenzene (DNCB), a model electrophile; and buthionine (S,R)-sulfoximine (BSO), an inhibitor of glutamate-cysteine ligase. NAPQI, DNCB and BSO can all cause glutathione (GSH) depletion; however only NAPQI and DNCB can covalently bind proteins. We also employed RNAi to manipulate Keap1 (the inhibitor of Nrf2), Nrf2 itself and NF-κB-p65, to understand their roles in the response to drug stress. All three chemicals induced Nrf2, but NF-κB binding activity was only increased after BSO treatment. In fact, NF-κB binding activity decreased after exposure to NAPQI and DNCB. While RNAi depletion of Keap1 led to reduced toxicity following exposure to DNCB, depletion of Nrf2 and NF-κB augmented toxicity. Interestingly, increased Nrf2 caused by Keap1 depletion was reversed by co-depletion of NF-κB. We demonstrate that Keap1/Nrf2 and NF-κB respond differently to electrophiles that bind proteins covalently and the redox perturbation associated with glutathione depletion, and that crosstalk may enable NF-κB to partly influence Nrf2 expression during cellular stress.

## Graphical abstract

CRMs activate Nrf2, but inhibit NF-κB, and GSH depletion without covalent modification activates both Nrf2 and NF-κB. This leads to cellular protection against the potentially harmful effects of redox perturbation.

## 1 Introduction

Drug-induced liver injury (DILI) is a major cause of hospital admissions [1], and one of the principal reasons for attrition of new chemical entities [2]. For example, acetaminophen (APAP), a commonly used analgesic known to induce liver injury on overdose, accounts for the most common form of acute liver failure in the United States [3]. A proportion of the pathogenesis of DILI may occur through the generation of chemically reactive metabolites (CRMs), usually formed through oxidative metabolism of drugs by phase 1 enzymes [2]. CRMs can covalently modify critical proteins, and bind to and deplete glutathione (GSH), the predominant cellular redox buffer. This causes disturbance to the cellular redox potential yielding a more oxidising environment [4]. Although there are defense mechanisms present to counter such disturbances [5], if the chemical challenge is overwhelming and the defense is breached, a switch from cell defense to cell death is favoured either by apoptosis or necrosis (for a review, see [2]). Thus, a cell will defend itself where possible, but under extreme conditions it may allow itself to die. The ability of a cell to respond to chemical stress occurs at least partially through the activation of transcription factors. These transcription factors are constitutively present in the cell or are rapidly synthesised when needed, and they facilitate the upregulation of proteins implicated in cell defense or cell death. Two redox-sensitive transcription factors, in particular, are the focus of much interest in determining the role of transcriptional adaption to chemical stress, namely nuclear factor-erythroid 2 (NF-E2)-related factor (Nrf2) and NF-kappa B (NF-κB).

Nrf2 plays a crucial role in cellular defense, and is the major regulator of anti-oxidant response elements (AREs) present in the regulatory region of the majority of cytoprotective genes [6]. Under basal conditions, Nrf2 is sequestered by Keap1 in the cytoplasm [7], and is constitutively targeted for ubiquitination by the Cul3-dependent E3 ubiquitin ligase complex, which results in its subsequent proteasomal degradation [8]. Under certain stress conditions, such as exposure to electrophiles or an increase in reactive oxygen species, it is believed that Keap1 undergoes a conformational change, blocking Nrf2 ubiquitination and enabling nuclear accumulation of Nrf2 and transactivation of ARE-dependent genes.

Nuclear factor kappa-light-chain-enhancer-of activated B cells (NF-κB) comprises five members belonging to the mammalian NF-κB family, i.e., p65 (Rel A), RelB, c-Rel, p50/p105 (NF-κB1) and p52/p100 (NF-κB2). NF-κB exists in the cytoplasm of unstimulated cells as homo- or hetero-dimers, bound to a family of inhibitory proteins known as IκB via non-covalent interactions. The predominant hetero-dimer present in cells is p65/p50 dimer [9]. Upon stimulation, the IκB kinase (IKK) complex which consists of a complex with two subunits, IKKα and IKKβ, and a regulatory subunit, NF-κB essential modifier (NEMO) [10], phosphorylates IκB-α [11]. Upon phosphorylation, IκB-α is targeted for ubiquitination and directed for proteasomal degradation [12]. This allows the release of NF-κB and its nuclear accumulation resulting in the transcription of genes involved in the inflammatory response, cell proliferation, cell survival and to a limited degree, the anti-oxidant response.

To date, there is little evidence for simultaneous regulation of Nrf2 and NF-κB within cells and little is known about the cellular consequences of such co-regulation. It may be valuable to understand what is happening to both transcription factors simultaneously to begin to understand the likely mechanisms that occur when a cell is exposed to chemical stress. The experiments presented here were designed to investigate the functional outcome of chemical stress/protein modification on the transcription factors as well being informative of how the cells actually sense these stresses. In this study, we hypothesised that the balance between both transcription factors might serve as a key cytoprotective mechanism to sense and respond to cell stress, where activation or inhibition of NF-κB, or Nrf2, or both transcription factors, may dictate the cellular response to stress.

## 2 Materials and methods

### 2.1 Materials

All chemicals and reagents were obtained from Sigma–Aldrich (Poole, UK), unless stated otherwise. Rabbit anti-mouse Nrf2 anti-body was a kind gift from Professor John Hayes (Biomedical Research Centre, University of Dundee).

### 2.2 Cell culture and treatment

Hepa-1c1c7 cells were maintained at 37 °C in a 5% CO_2_ atmosphere in Dulbecco's modified Eagle's medium (DMEM; Lonza, Wokingham, UK) supplemented with 584 mg/L l-gutamine, 10% fetal bovine serum (FBS), 100 U/ml penicillin, and 100 μg/ml streptomycin. For all treatments, cells were incubated with serum-free DMEM containing the indicated compound. All compounds were dissolved in dimethyl sulfoxide (DMSO) at a final concentration of the solvent in media of 0.1%, except for buthionine sulfoximine (BSO), which was dissolved in water.

### 2.3 RNA interference

Short interfering RNA (siRNA) duplexes targeting mouse Keap1, and Nrf2 were purchased from Dharmacon (Lafayette, CO, USA) and molecules targeting p65 were purchased from Invitrogen (Paisley, UK). The siRNA duplexes were as follows; si-Nrf2 sense 5′-GCA AGA AGC CAG AUA CAA A-3′, anti-sense 5′-U UUG UAU CUG GCU UCU UGC-3′, si-Keap1 sense 5′-GCU AUG ACC CGG ACA GUG A-3′, anti-sense 5′-U CAC UGU CCG GGU CAU AGC-3′, si-p65 sense 5′-UUC AUC UCC GGA GAG ACC AUU GGG A-3′, anti-sense 5′-U CCC AAU GGU CUC UCC GGA GAU GAA-3′. Hepa-1c1c7 were plated out onto 96-well plates at 1 × 10^4^ cells/well for NF-κB siRNA validation, 96-well plates at 7 × 10^3^ cells/well for LDH assay, 24-well plates at 5 × 10^4^ cells/well for GSH assays and 10-cm dishes at 4 × 10^6^ cells/dish for nuclear extractions. Cells were transfected with 10 nM of siRNA directed against Keap1 or Nrf2, and 3 nM siRNA against p65 for 48 h, using Lipofectamine 2000 (Invitrogen, Paisley, UK) according to the manufacturer's instructions.

### 2.4 Preparation of cytosolic and nuclear extracts and whole cell lysates

For experiments with nuclear proteins, cells were treated with NF-κB siRNA for 48 h or treated for 1 h with the chemicals under investigation. Cytosolic and nuclear extracts were prepared using the method of Dignam et al. [13]. For whole cell lysate experiments, cells were treated with siRNA for 48 h and 6 h with DMSO. After treatments, cells were lysed with radioimmunoprecipitation assay buffer. Total protein content was measured according to the method of Bradford protein assay [14] with commercial kit (Bio-Rad, Hemel Hempstead, UK) and samples were stored at −80 °C before analysis.

### 2.5 Western immunoblotting

Cytosolic or nuclear extracts or whole cell lysates were resolved by denaturing electrophoresis on pre-cast 4–12% Novex bis–tris polyacrylamide gels (Invitrogen), using a 3-(N-morpholino)propanesulfonic acid running buffer. Separated proteins were transferred to Hybond nitrocellulose membranes (GE Healthcare Life Sciences, Little Chalfont, UK), which were blocked for 15 min in 10% non-fat milk (Bio-Rad) in Tris-buffered saline (TBS, pH 7.0) containing 0.1% Tween 20. Blocked membranes were probed with rabbit anti-mouse Nrf2 (1:5000) in 10 ml of 2% bovine serum albumin (BSA) for 1 h. After several washes, membranes were probed with goat anti-rabbit (1:10000) horseradish peroxidase-conjugated anti-IgG for 1 h. Western blots were visualised using enhanced chemiluminescence (PerkinElmer, Beaconsfield, UK) and Hyperfilm (GE Healthcare Life Sciences). All blots were also probed for actin (1:5000) as a loading control. Recombinant mouse His-tagged Nrf2 (+con) was loaded as a standard to confirm anti-body specificity. Western blot band volumes were quantified using TotalLab 100 software (Nonlinear Dynamics, Newcastle, UK) and normalised against actin.

### 2.6 NF-κB electrophorectic mobility shift assay (EMSA)

An NF-κB wild-type oligonucleotide (Wt) probe 5′ to 3′ strand: (5′-AGT TGA GGG GAC TTT CCC AGG C-3′) was 5′ end-labelled with adenosine 5′-triphosphate [γ-32P] ATP (PerkinElmer Life science, Mechelen, Belgium) using T4 polynucleotide kinase (Promega Corp, Madison WI, USA). Nuclear extracts (5 μg) were incubated with labelled oligonucleotide in a binding buffer containing (0.1 μg/μl) polydIdC (Amersham), 4% Ficoll, 20 mM HEPES, 35 mM NaCl, 60 mM KCl, NP40 0.01% and 2 mM dithiothreitol (DTT) for 20 min at room temperature. The definition of a NF-κB specific shift was made according to the following criteria; supershift assays using NF-κB specific anti-bodies, addition of mouse anti-p65 and/or anti-p50 (Santa Cruz) and a control non-specific anti-body. For competition assays, unlabelled wild-type or mutant (5′-AGT TGA GG***C*** GAC TTT CCC AGG C-3′) oligonucleotides were added in excess for 10 min prior to addition of labelled oligonucleotide. Protein–DNA complexes were resolved by non-denaturing electrophoresis on a 5% polyacrylamide gel for 1.5 h at 200 V in 0.5× Tris–Borate–EDTA buffer. Gels were subsequently dried and exposed to a Phosphor Imager screen (Molecular Dynamics, Sunnyvale, CA). The gels were visualised and bands were quantified using ImageQuant software (Molecular Dynamics).

### 2.7 Confocal microscopy

Hepa-1c1c7 cells were plated out on Lab-Tek II chamber slides (Nalge Nunc, Rochester, NY) at 2.5 × 10^5^ cells/chamber for 24 h. Following treatment, cells were washed in phosphate buffered saline (PBS) and fixed in 4% paraformaldehyde at 4 °C for 30 min. Fixed cells were permeabilised with 0.2% Triton X-100, quenched with 100 mM glycine and blocked with 10% FBS, for 10 min each. Cells were then incubated with rabbit anti-mouse Nrf2 or monoclonal anti-mouse p65 (1:500) in 2% FBS at 37 °C for 1 h. Following several washes in PBS, cells were incubated with FITC-conjugated goat anti-rabbit or FITC-conjugated goat anti-mouse (1:250) in 2% FBS at 37 °C for 1 h. Cells were washed several times with PBS and nuclei were counter-stained with Hoechst 33258 (2 μg/ml) (Invitrogen) in PBS at room temperature for 10 min. Chambers were detached from the slides and coverslips were mounted using Vectashield hard-set medium (Vectorlabs, Peterborough, UK). Immunofluoresence was visualised using a Leica SP2 AOBS confocal microscope (Leica Microsystems, Milton Keynes, UK).

### 2.8 Measurement of lactate dehydrogenase leakage

Hepa-1c1c7 cells were plated out on 96-well plates at 2 × 10^4^ cells/well for 24 h. Following treatment, lactate dehydrogenase (LDH) leakage was measured using a Cytotoxicity Detection Kit (Roche Applied Science, Burgess Hill, UK) in accordance with the manufacturer's instruction. LDH leakage from cells into the culture medium (extracellular) is expressed as a percentage of total LDH (intracellular plus extracellular).

### 2.9 Measurement of glutathione

Hepa-1c1c7 cells were plated out on 24-well plates at 2 × 10^5^cells/well for 24 h. Total GSH content was quantified using the 5,5-O-dithiobis(2-nitrobenzoic acid) –GSH reductase recycling method, as previously described by Vandeputte et al. [15]. Sample GSH concentrations were calculated via reference to a standard curve ranging from 0 to 50 nmol/ml GSH. The GSH concentration for each sample was normalised to total protein content.

### 2.10 Data analysis

Data are expressed as mean ± standard deviation of the mean. The significance of differences within the data was assessed by Kruskal–Wallis analysis of variance (ANOVA), one-way ANOVA or Student's *t*-test. A difference was considered significant at *p* < 0.05.

## 3 Results

### 3.1 N-acetyl-p-benzoquinoneimine (NAPQI) and dinitrochlorobenzene (DNCB) activate Nrf2 but inhibit NF-κB activity

**Fig. 1:**
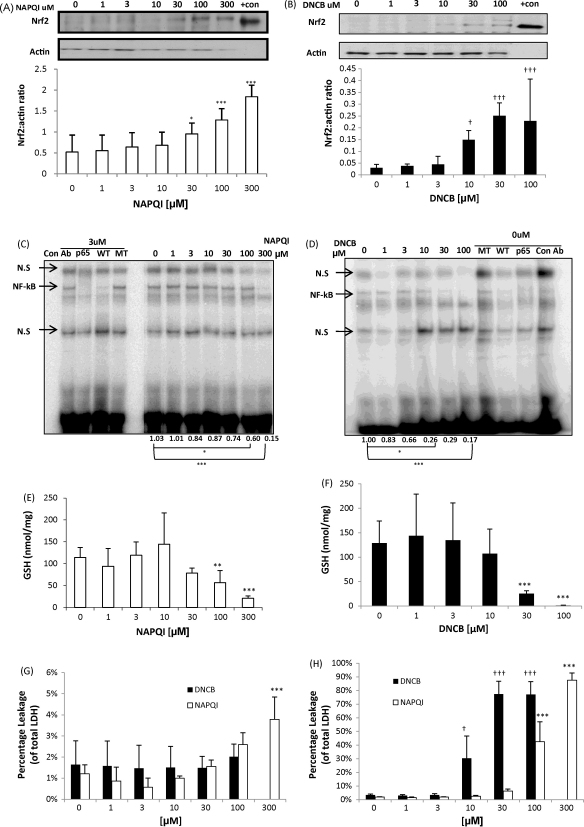
Chemical stress activates Nrf2 and inhibits NF-κB. Cells were treated for 1 h with NAPQI (A) or DNCB (B) and nuclear protein resolved on SDS-PAGE and probed for Nrf2. NAPQI (A) and DNCB (B) activates Nrf2 nuclear accumulation dose-dependently. Actin is shown as loading control. Recombinant mouse His-tagged Nrf2 was loaded as a positive control on the blot (+con). The bottom panel shows the densitometric analysis of Nrf2 positive bands normalised against actin. Paired *t*-test, *p < 0.05, ***p < 0.001. Kruskal–Wallis, ^†^*p* < 0.05, ^†††^*p* < 0.001. Cells were treated for 1 h with NAPQI (C) or DNCB (D) and nuclear protein was assayed using EMSA for NF-κB activity. NAPQI (C) and DNCB (D) inhibit NF-κB binding activity dose-dependently. The assay was specific for the NF-κB protein as addition of a 50-fold excess of wild-type oligonucleotide (WT) to the reaction displaced the bound protein from the radiolabelled oligonucleotide, while this was not observed with the addition of excess mutant (MT) oligonucleotide. The addition of specific anti-NF-κB anti-body (p65), as opposed to non-specific anti-body (con Ab) to the binding reaction caused a supershift in the NF-κB band confirming the composition of the NF-κB complex. The data demonstrate the densitometic analysis of NF-κB normalised against control. Kruskal–Wallis, *p < 0.05, ***p < 0.001. Cells were treated for 1 h with NAPQI (E) or DNCB (F) and lysed with 10 mM HCL. GSH depletion reaches 80% of control with the highest dose of NAPQI (E) and 100% of control with highest dose of DNCB (F). Total GSH normalised against total protein content. Kruskal–Wallis, **p < 0.01, ***p < 0.001. Cells were treated for 1 h with NAPQI or DNCB (G) and 24 h with NAPQI (H). LDH assay shows minimal leakage upon treatment with NAPQI (white) or DNCB (black) for 1 h (G). LDH assay shows substantial leakage upon treatment with NAPQI (white) or DNCB (black) (H) after 24 h. Extracellular LDH activity is expressed as a percentage of total (extracellular plus intracellular) LDH activity. One-way ANOVA, ***p < 0.001, ^†^*p* < 0.05, ^†††^*p* < 0.001. In all experiments, the control cells were treated with 0.1% DMSO.

In common with all mammalian hepatoma cell lines, Hepa-1c1c7, lacks metabolic competence and therefore cannot directly bioactivate acetaminophen. Consequently, NAPQI, the chemically reactive metabolite of acetaminophen [16], was used in these studies along with DNCB, a model alkylating agent and contact sensitizer [17]. Both have been previously shown to deplete GSH and covalently bind cellular proteins [18–22]. Following a 1 h exposure, nuclear accumulation of Nrf2 increased with increasing concentrations of NAPQI (Fig. 1A) and DNCB (Fig. 1B); an increase in Nrf2 after exposure to NAPQI or DNCB has been demonstrated previously in our lab [18]. Immunochemical analysis of Nrf2 and NF-κB-p65 cellular localisation shows an increase in NF-κB-p65 cellular abundance (Fig. 3A, second panel) and a clear increase in Nrf2 (Fig. 3B, second panel) accumulation in the nucleus after 1 h of DNCB treatment. These cells consistently express low but detectable levels of NF-κB DNA-binding activity (Fig. 1C and D), however on the contrary to Nrf2 expression, NF-κB DNA binding decreased with increasing concentrations of NAPQI (Fig. 1C) and DNCB (Fig. 1D). Both chemicals caused a depletion of total GSH, which fell to 20% of the control at the highest dose of NAPQI (Fig. 1E) and to undetectable levels at the highest dose of DNCB (Fig. 1F). Lactate dehydrogenase (LDH) leakage assays show limited leakage after exposure of cells to test compounds for 1 h, although this is significant at 300 μM of NAPQI (Fig. 1G). The assay demonstrates substantial toxicity at 24 h following exposure to concentration of NAPQI at 100 and 300 μM, and with DNCB at all concentration between 10 and 100 μM (Fig. 1H).

### 3.2 Glutathione depletion activates both Nrf2 and NF-κB activities

**Fig. 2:**
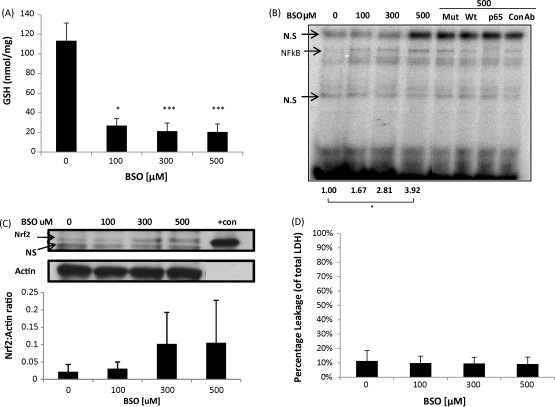
Depletion of glutathione activates Nrf2 and NF-κB. Cells were treated for 24 h with BSO and lysed with 10 mM HCL. GSH was depleted by a maximum of 80% of control (A). Total GSH normalised against total protein content. Kruskal–Wallis, *p < 0.05, ***p < 0.001. Cells were treated for 24 h with BSO and nuclear protein was assayed for NF-κB activity. BSO activates the translocation and binding of NF-κB dose-dependently (B). The assay was specific for the NF-κB protein as addition of a 50-fold excess of wild-type oligonucleotide (Wt) to the reaction displaced the bound protein from the radiolabelled oligonucleotide, while this was not observed with the addition of excess mutant (Mut) oligonucleotide. The addition of specific anti-NF-κB anti-body (p65), as opposed to non-specific anti-body (con Ab) to the binding reaction caused a supershift in the NF-κB band confirming the composition of the NF-κB complex. The data shows the densitometric analysis of NF-κB normalised against control. Kruskal–Wallis, *p < 0.05. Cells were treated for 24 hs with BSO and nuclear protein resolved on SDS-PAGE and probed for Nrf2. BSO activates Nrf2 nuclear accumulation dose-dependently (C). Actin is shown as loading control. Recombinant mouse His-tagged Nrf2 was loaded as a positive control on the blot (+con). The bottom panel shows densitometric analysis of Nrf2 normalised against actin. Cells were treated for 24 h with BSO. LDH assay shows minimal leakage upon treatment with BSO (D). Extracellular LDH activity is expressed as a percentage of total (extracellular plus intracellular) LDH activity. In all experiments, the control cells were treated with 0.1% DMSO.

**Fig. 3:**
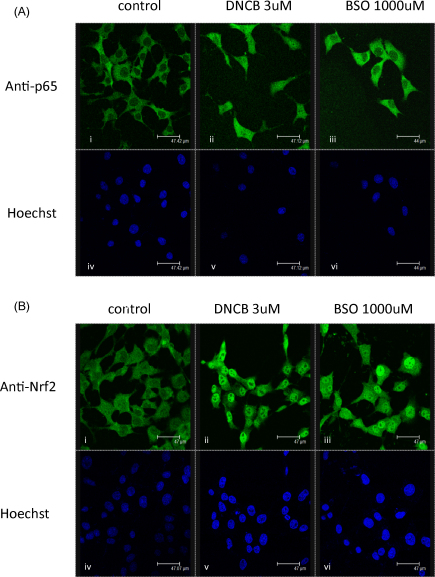
Subcellular localisation of p65 (A) and Nrf2 (B) in Hepa-1c1c7 cells exposed to control and DNCB or BSO for 1 and 24 h, respectively. FITC-conjugate goat anti-p65 (A)(i, ii and iii), FITC-conjugate goat anti-Nrf2 (B)(i, ii and iii) and Hoechst 33258 (A)(B)(iv, v and vi) immunofluorescence were visualised by confocal microscopy.

We then examined whether GSH depletion and/or the protein covalent modification due to the electrophilic nature of these compounds was responsible for the different effects observed on these pathways. We therefore used l-buthionine(S,R)-sulfoximine (BSO), which depletes intracellular GSH efficiently by irreversible inhibition of the rate-limiting enzyme of GSH synthesis, GCLC [23]. It is now well-accepted that depletion of GSH due to exposure of cells to BSO leads to redox perturbation in a variety of cell models, for recent examples see [24,25]. BSO concentrations of 3 to 30 μM depletes GSH after 24 h exposure (Supplementary Fig 1A), but does not induce NF-κB binding activity (Supplementary Fig 1B). However, 100–500 μM of BSO increased NF-κB binding activity (Fig. 2B) and increased nuclear accumulation of Nrf2 (Fig. 2C), which correspond with GSH levels being depleted to below 20% of the basal level (Fig. 2A); a BSO-mediated increase in Nrf2 has been seen previously in our lab [18]. Immunochemical analysis of Nrf2 and NF-κB cellular localisation after 24 h of BSO exposure indicates some cellular accumulation of NF-κB (Fig. 3A, third panel) and substantial nuclear accumulation of Nrf2 (Fig. 3B, third panel). 24 h exposure to BSO does not cause toxicity in the cells (Fig. 2E).

### 3.3 Comparison of Nrf2 and NF-κB regulation by NAPQI, DNCB and BSO

**Fig. 4:**
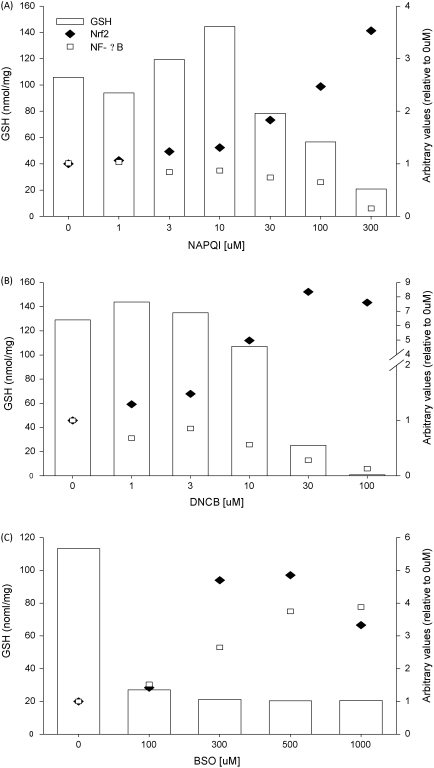
Effect on Nrf2, NF-κB and GSH by treatment of cells with NAPQI, DNCB and BSO. NAPQI (A) and DNCB (B) increased Nrf2 nuclear accumulation and decreased NF-κB binding activity concurrently with GSH depletion. BSO (C) increased both Nrf2 nuclear accumulation and NF-κB binding activity concurrently with GSH depletion. Error bars have been omitted from graphs for visual clarity.

The effect of treatment with NAPQI, DNCB or BSO on Nrf2 and NF-κB activity can be seen clearly in double-y graphs (Fig. 4). NAPQI (Fig. 4A) and DNCB (Fig. 4B), which can covalently bind proteins and deplete GSH, increased Nrf2 activity and decreased NF-κB binding activity, while the GSH-depleting agent, BSO (Fig. 4C), increased both Nrf2 and NF-κB activity.

### 3.4 RNA interference modulates Keap1, Nrf2 and NF-κB, p65 subunit expression

RNAi is a conserved mechanism of specific gene silencing through posttranslational degradation of mRNA [26]. In this study, we used RNAi to investigate the physiological and molecular effects of Keap1, Nrf2 and p65 silencing on cellular cytoprotection. We have already depleted Keap1 and Nrf2 with RNAi, and the molecules used resulted in 80% silencing of each protein [18]. RNAi directed against NF-κB, p65 subunit expression was also successful. A 70% reduction in basal NF-κB p65 expression was detected in cells treated with p65 RNAi (Supplementary Fig 2A) concurrently with a decrease in nuclear NF-κB DNA-binding activity (Supplementary Fig 2B).

### 3.5 RNAi modulation of Nrf2, Keap1 and p65 influences cell defense in hepa-1c1c7 cells

**Fig. 5:**
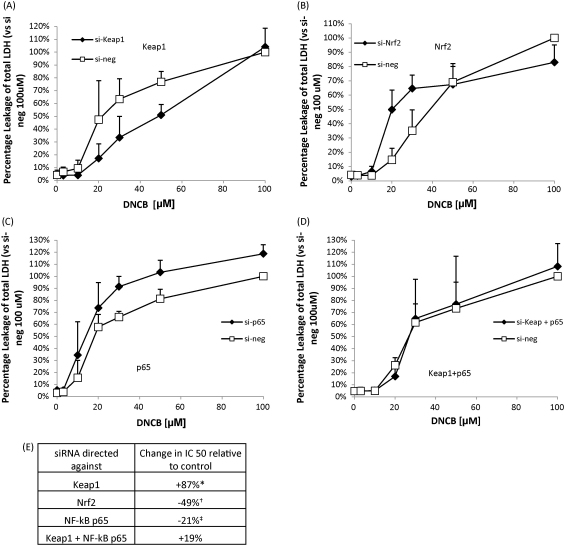
RNAi modulation of Nrf2, Keap1 and p65 influences cell defense. Cells were transfected for 48 h with RNAi and treated for 6 h with DNCB. Keap1 depletion decreased DNCB toxicity (A). Nrf2 (B) and p65 (C) depletion increased DNCB toxicity. Keap1 and p65 co-depletion shows the effect of Keap1 knock down alone is reversed by depletion of p65 (D). The change in IC50 of each RNAi treatment is expressed in (E). Extracellular LDH activity is expressed as a percentage of total (extracellular plus intracellular) LDH activity versus si-neg control treated with 100uM DNCB. All data compared to their respective negative controls. Paired *t*-test, *p < 0.05, si-keap1 versus si-neg, ^†^*p* < 0.05 si-Nrf2 versus si-neg and ^‡^*p* < 0.05 si-p65 versus si-neg. In all experiments, the control cells were treated with 0.1% DMSO.

Modulation of Nrf2, Keap1 and NF-κB, p65 had a profound effect on the ability of Hepa-1c1c7 cells to elicit a defense response against chemical stress. DNCB was again used as a model electrophile to induce chemical stress. LDH assays indicated that RNAi knock down of Keap1 (Fig. 5A) protects the cells against DNCB toxicity and shows that DNCB toxicity was augmented when Nrf2 was depleted with RNAi directed against Nrf2 (Fig. 5B) and p65 was depleted (Fig. 5C) individually. Interestingly, the phenotypic effect of the Keap1 knock down was reversed when both Keap1 and p65 (Fig. 5D) were depleted simultaneously. The IC50s of the siRNA treatments compared to their respective negative controls show that Keap1 silencing causes a 20 μM increase in IC50, Nrf2 silencing causes a 18uM decrease in IC50, si-p65 causes a 4 μM decrease in IC50 and si-Keap1 + p65 knock down leads to a 5 μM decrease in IC50 (Fig. 5E).

### 3.6 RNAi depletion of Nrf2 and Keap1, but not p65, influences the basal level of GSH in hepa-1c1c7 cells

**Fig. 6:**
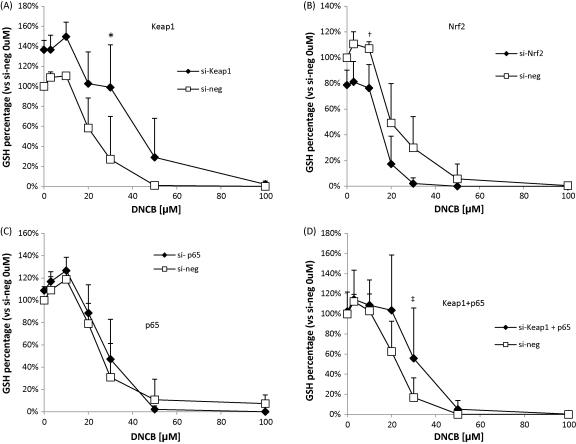
RNAi modulation of Nrf2, Keap1 and p65 influences the level of GSH. Cells were transfected for 48 h with RNAi and treated for 6 h with DNCB. Keap1 depletion increases basal GSH levels (A). Nrf2 depletion caused a decrease in basal GSH level (B). With p65 depletion there is no change in GSH level basally (C). Where of both Keap1 and p65 are depleted simultaneously, there is no increase in basal GSH levels (D). Total GSH normalised against total protein content versus si-neg control untreated. All data are compared to their respective negative controls (si-neg). Kruskal–Wallis ANOVA, *p < 0.05 si-keap1 versus si-neg, ^†^*p* < 0.05 si-Nrf2 versus si-neg and ^‡^*p* < 0.05 si-Keap1 + p65 versus si-neg. In all experiments, the control cells were treated with 0.1% DMSO.

We then measured the effect of modulation of Nrf2, Keap1 and p65 on the basal level of GSH to determine whether the GSH level has an influence on the toxicity seen with DNCB. Keap1 silencing increased the basal level of GSH by approximately 40% and at 10 μM of DNCB, the GSH level increased by an additional 20% (Fig. 6A). Nrf2 silencing decreased the basal level of GSH by approximately 20% and at 20μM of DNCB, there is a clear decrease in GSH levels to less than 20% of total GSH (Fig. 6B). p65 RNAi did not affect the basal level of GSH or levels after treatment with DNCB (Fig. 6C). Interestingly, in the presence of Keap1 and NF-κB-p65 RNAi, there is no increase in the basal GSH level (Fig. 6D).

### 3.7 Simultaneous depletion of Keap1 and NF-κB-p65 by RNAi influences the basal level of Nrf2 in hepa1c cells

**Fig. 7:**
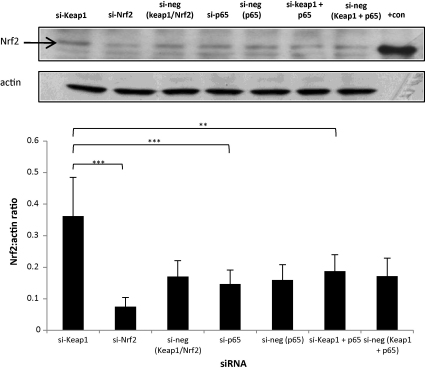
Simultaneous RNAi modulation of Keap1 and p65 influences the level of Nrf2 protein. Cells were transfected for 48 h with RNAi. Whole cell lysates were resolved on SDS-PAGE and probed for Nrf2. Keap1 depletion increases basal Nrf2 and the depletion of Nrf2 decreases basal Nrf2. p65 depletion does not change Nrf2 basally in the cells. The depletion of Keap1 and p65 simultaneously reduces the expression of Nrf2 basally. All data are compared to their respective controls. Actin is shown as loading control. Recombinant mouse His-tagged Nrf2 was loaded as a positive control on the blot (+con). The bottom panel shows the densitometric analysis of Nrf2 normalised against actin. One-way ANOVA, **p < 0.01, ***p < 0.001.

In order to investigate the possible influence of NF-κB-p65 on the activity of Nrf2, the effect of RNAi depletion of NF-κB-p65 on Nrf2 expression was determined in Hepa-1c1c7 cells. p65 RNAi alone had no effect on the cellular expression of Nrf2, however, when co-transfected with Keap1 RNAi, p65 depletion was able to reverse the induction of Nrf2 caused by Keap1 depletion (Fig. 7). This suggests that p65 plays a role in the modulation of Nrf2 expression.

## 4 Discussion

The roles of the NF-κB pathway in cellular survival, proliferation and the inflammatory response and of the Nrf2/Keap1 pathway in the cellular defense against chemical stress and redox perturbation are well described. However, it is still unclear as to how both pathways simultaneously respond to these latter insults. In our study, both chemical stress and GSH depletion with associated redox perturbation induced Nrf2, detected as nuclear accumulation observed by confocal microscopy and Western blotting (Fig. 3). Chemical stress induced by exposure to DNCB caused a decrease in NF-κB binding activity (Fig. 1D) despite the cellular accumulation of NF-κB-p65 (Fig. 3A). On the other hand, redox perturbation that ensues from the GSH-depleting action of BSO activates nuclear translocation of Nrf2 as well as causing increased DNA-binding activity of NF-κB (Figs. 1 and 2). This is consistent with the notion that Nrf2 can be activated through covalent modification as well as GSH depletion. In the case of NF-κB, activity can either be enhanced by GSH depletion, or inhibited by reactive chemical exposure, through cysteine modification by covalent binding to one or more subunits in NF-κB, or to other factors that regulate NF-κB. Here we have observed that NAPQI and DNCB inhibits NF-κB DNA activity, but the question as to whether both chemicals control NF-κB through an upstream effect or by directly interfering with its activity is unclear and hence further investigation is needed. For both transcription factors, nevertheless, it should be noted that a definitive link between critical cysteine(s) modification, which may be irreversible or reversible, as a consequence of covalent binding or GSH depletion, and a change in activity still remains to be determined experimentally.

Our observations are in agreement with previous studies in animal and cell-based models that showed that NAPQI induces the activation of Nrf2 [18,27] and inhibits NF-κB DNA-binding activity [28]. BSO depletes GSH by binding to the active site of GCLC non-covalently, inhibiting the activity of GCLC [23]. In our cell-based model, we have shown that depletion of GSH by BSO induces the nuclear accumulation of Nrf2 and increases NF-κB binding concurrently (Fig. 2). The depletion of GSH over 24 h by BSO does not elicit cytotoxicity (Fig. 2D). This is in agreement with our previous finding in which BSO induced nuclear accumulation of Nrf2 [18] and findings by others in which BSO induced NF-κB activity [29,30]. It should be emphasized that the induction of Nrf2 and NF-κB by BSO only occurs if the depletion of GSH is to below 20% of total cellular GSH at 100 μM, which may be the threshold level to induce both transcription factors. In addition, both of the Nrf2 and NF-κB responses to NAPQI, DNCB and BSO occur at concentrations where toxicity is not or has not yet become apparent, hence these factors appear to be able to sense and respond before toxicity emerges.

It has been shown previously that 15-deoxy-Δ12, 14-prostaglandin J2 (15d-PG J2), an endogenous cyclopentenone prostaglandin molecule which can exert a powerful anti-inflammatory activity, is able to induce Nrf2/Keap1 and inhibit NF-κB [31,32]. While 15d-PGJ2 is able to directly bind to Keap1 thiol residues and induce Nrf2, without the depletion of GSH [18,33], NF-κB binding activity can be inhibited by 15d-PGJ2 through the direct modification of the p65 subunit [32]. Therefore, it is becoming clear that exogenous or endogenous compounds possessing the potential for protein covalent binding display very different effects on the Nrf2/Keap1 and NF-κB pathways, whereas agents such as BSO that deplete GSH– may have similar effects on both redox-sensitive transcription factors. Hence, these factors may serve as sensors to different types of stress, allowing cells to adapt and respond in a different but appropriate manner.

An important issue that needs to be considered regarding the validity of experiments such as those in the present study, is the fact that a reactive metabolite (in this case NAPQI) is added exogenously to cells rather than being generated endogenously. The necessity to add a reactive metabolite outside of the cell remains a major hurdle in attempting to study the molecular pathways elicited by exposure of cells to drugs that require bioactivation. Nevertheless, data from this laboratory addressing the reactivity of residues in several proteins (i.e. GST-P and Keap1 – the regulator of Nrf2) show that these proteins demonstrate similar profiles of cysteine reactivity, as assessed by mass spectrometry, whether the protein is present at endogenous levels (GST-P) or is over-expressed (Keap1), to the profile of NAPQI binding to the recombinant proteins *in vitro* [18,34]. Therefore, while there are certainly limitations that need to be considered when a reactive metabolite is added exogenously to a cell rather than generated intracellularly, for certain reactive metabolites the profiles of modification outside and inside a cell may be similar.

Both Nrf2 and NF-κB play important roles in cellular defense, by inducing anti-oxidant enzymes, phase II enzymes and anti-apoptotic proteins. Co-regulation has not been shown before, therefore we have attempted to explore the consequences of such co-regulation by studying the effect of modulating the level of each transcription factors pathway both separately and simultaneously. Using RNAi with DNCB as a model electrophile, we show here that an increase in cellular Nrf2, caused by Keap1 silencing, shifts the DNCB toxicity curve to the left, demonstrating increased resistance to DNCB toxicity. Conversely, decreased Nrf2 protein expression resulted in an increased susceptibility to DNCB toxicity. These findings support previous data [18] and emphasize the importance of Nrf2 in cellular defense. From our data, p65 does not appear to play a significant role in cell protection since si-p65 reduced the DNCB IC50 by only 4 μM. To better understand the role of each transcription factor individually and in concert with respect to cell defense and protection, we used RNAi simultaneously against Keap1 and p65, in order to: (1) monitor how changes in one transcription factor pathway alters the response mediated by the other pathway and (2) to attempt to model using this molecular genetics approach what we have observed in the cell during the response of Nrf2 and NF-κB to chemical stress elevated by DNCB. As predicted, depletion of Keap1, the inhibitor of Nrf2, increased cellular defense. The co-transfection of RNAi against Keap1 and p65 reversed the phenotypic effect seen with individual Keap1 knock down (Fig. 5D). The protective effect of Nrf2 induction caused by the loss of Keap1 was reduced when p65 was depleted, implying a role for p65 as a modulator of Nrf2-dependent cell protection. Therefore, our data suggest that modulation of p65 has an effect on the ability of the cell to defend against toxicity via the Nrf2/Keap1 pathway, and that the Nrf2 and NF-κB pathways are linked.

GSH is a major anti-oxidant that quenches reactive xenobiotics and endogenous oxidative stress. Therefore, we examined if the depletion of Keap1, Nrf2 and p65 has any effect on the basal level of GSH thus further influencing the cells’ ability to defend against electrophile toxicity. We have demonstrated that the depletion of Keap1 increases the basal level of GSH (Fig. 6A), and depletion of Nrf2 decreases the basal level of GSH (Fig. 6B). This is consistent with the evidence that Nrf2 is one of the main regulators of GSH synthesis [18,35–37]. RNAi depletion of p65 has no effect on the level of GSH basally or in the presence of DNCB (Fig. 6C). We have shown that co- modulation of Keap1 and p65 affects the cell's ability to defend against DNCB toxicity. Interestingly, the simultaneous depletion of p65 and Keap1 prevented the increase in basal levels of GSH (Fig. 5D) seen with Keap1 knock down only. In line with this we have shown that the expression of Nrf2 was reduced by the co-depletion of Keap1 and p65 (Fig. 7). The decrease in basal Nrf2 protein expression may explain the reverse in DNCB toxicity protection and the reduction of basal GSH by Keap1 depletion. Therefore, these observations imply that p65 may play an important role in the expression and the function of Nrf2 and further work is required to fully test this hypothesis.

The results of this study, indicating crosstalk between the Nrf2 and NF-κB pathways support evidence from other groups demonstrating that the GSH rate-limiting synthetic enzyme, GCLC, is regulated by Nrf2 indirectly through NF-κB [38–40]. Recently, it has also been shown that Keap1 is able to interact directly with IKKβ and to promote its proteasomal degradation via ubiquitination in human cells [41]. In this study, overexpression of Keap1 was seen to repress TNF-α induced NF-κB activity, while the depletion of Keap1 by RNAi induced the nuclear accumulation and activity of NF-κB. This suggests that Keap1 not only plays an important role in regulating Nrf2 but may also be a key regulator of the NF-κB pathway.

**Fig. 8:**
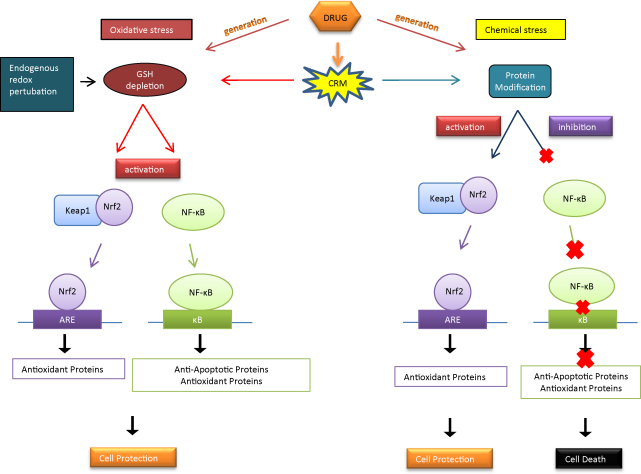
Proposed regulation of Nrf2 and NF-κB by protein modification or GSH depletion. (This scheme is based upon data derived in this study and other work ([18,27–30,42])). Protein covalent modification associated with exposure to chemically reactive metabolites activates Nrf2 inducing the transcription of cytoprotective proteins. However, protein covalent modification inhibits NF-κB, thereby reducing the transcription of anti-apoptotic and anti-oxidant proteins. This leads to cellular protection through the Nrf2 pathway, but if the protection is overwhelmed, cell death may ensue due to inhibition of NF-κB. GSH depletion without covalent modification will cause intracellular redox perturbation, activation of both Nrf2 and NF-κB, inducing the transcription of cytoprotective proteins, and anti-apoptotic and anti-oxidant proteins, respectively. This may lead to cellular protection against the potentially damaging effects of redox perturbation.

In conclusion, we have demonstrated that the transcription factors, Nrf2/Keap1 and NF-κB, respond differently to arylating and GSH-depleting agents. During chemical stress, it is likely that the cell attempts to protect itself by activating Nrf2, inducing a pleiotropic transcriptional response of cytoprotective proteins. However, when this protective mechanism is overwhelmed and cellular damage is too great, it may be more beneficial for the cells to undergo apoptotic cell death due to the inhibition of NF-κB. During redox perturbation, to which the cell is constantly exposed under physiological conditions, activation of both Nrf2 and NF-κB may occur to protect the cells and prevent further damage (Fig. 8). Nonetheless, protein modification by chemical arylation or oxidation as a consequence of GSH depletion on other signal transduction pathways involved in cellular protection or toxicity needs to be examined. Also, the use of GSH modulators such as N-acetylcysteine and GSH ester, or GSH depletors such as diethylmaleate and phorone, and the measurement of oxidative stress using chemical probes should be considered in future investigations. Our results also suggest a crosstalk between Nrf2/Keap1 and NF-κB pathways, where p65 was shown to modulate the expression and functionality of Nrf2. More work is required to fully define the emerging links between the Nrf2/Keap1 and NF-κB pathways, and how these impact on cellular response to drug exposure.

## Appendix A Supplementary data

*Supplementary Fig. 1*: BSO treatment of Hepa1c. Cells were treated for 24 h with BSO and lysed with 10 mM HCL. GSH was depleted by BSO (A). Total GSH normalised against total protein content. One-way ANOVA, ^†††^*p* < 0.001. Cells were treated for 24 h with BSO and nuclear protein was assayed for NF-κB activity (B). The assay was specific for the NF-κB protein as addition of a 50-fold excess of wild-type oligonucleotide (Wt) to the reaction displaced the bound protein from the radiolabelled oligonucleotide, while this was not observed with the addition of excess mutant (Mut) oligonucleotide. The addition of specific anti-NF-κB anti-body (p50, p65), as opposed to non-specific anti-body (con Ab) to the binding reaction caused a supershift in the NF-κB band confirming the composition of the NF-κB complex.

*Supplementary Fig. 2*: RNAi modulates NF-κB, p65 subunit expression. Cells were transfected for 48 h with NF-κB siRNA and nuclear protein resolved on SDS-PAGE and probed for NF-κB. NF-κB siRNA deplete NF-κB protein expression to approximately 70% of control (A). Actin is shown as loading control. Cell lysates from HepG2 over-expressed p65 was loaded as positive control on the blot (+con). si-negM and si-negL are controls for si-p65#1/si-p65#2 and si-p65#3, respectively. The bottom panel shows densitometric analysis of NF-κB positive bands normalised against actin. Paired *t*-test, **p* < 0.05. Cells were transfected for 48 h with NF-κB siRNA and nuclear protein was assayed for NF-κB activity. NF-κB siRNA decreases constitutive binding compared to control (B). The assay was specific for the NF-κB protein as addition of a 50-fold excess of wild-type oligonucleotide (Wt) to the reaction displaced the bound protein from the radiolabelled oligonucleotide, while this was not observed with the addition of excess mutant (Mut) oligonucleotide. The addition of specific anti-NF-κB anti-body (p50, p65), as opposed to non-specific anti-body (con Ab) to the binding reaction caused a supershift in the NF-κB band confirming the composition of the NF-κB complex. The data shows the densitometric analysis of NF-κB normalised against control.
