# Supplementary figures and images for: Differential effect of covalent protein modification and glutathione depletion on the transcriptional response of Nrf2 and NF-κB
Source: Biochem Pharmacol. 2010 Aug 1;80(3):410–21. doi: 10.1016/j.bcp.2010.04.004 (PMC2884179; doi:10.1016/j.bcp.2010.04.004)

Supplementary Figures

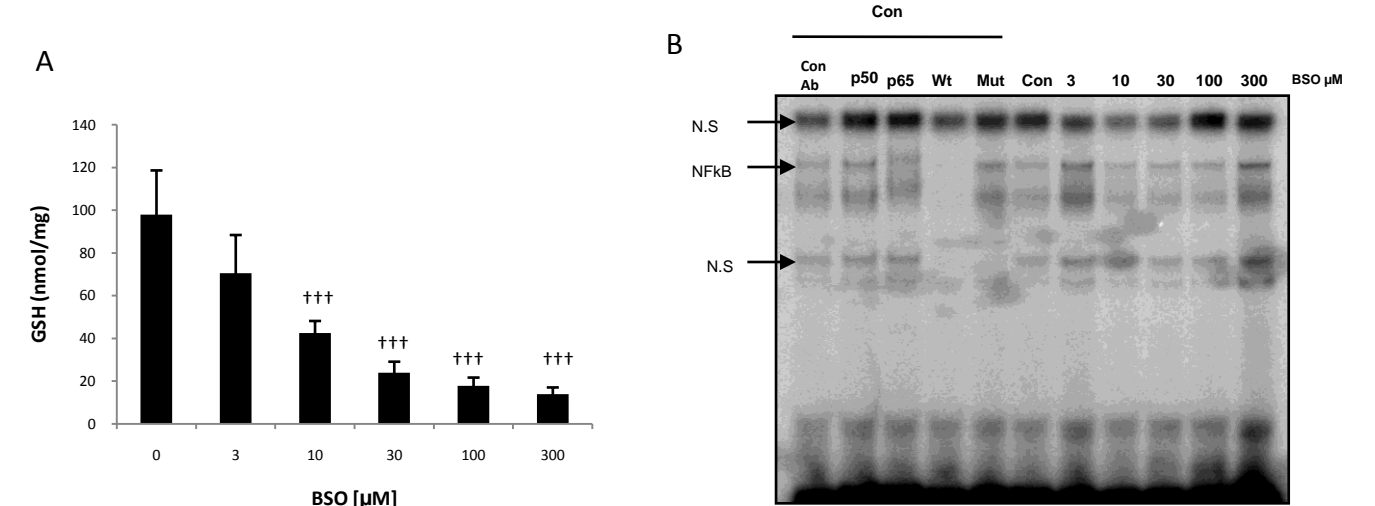

Fig 1

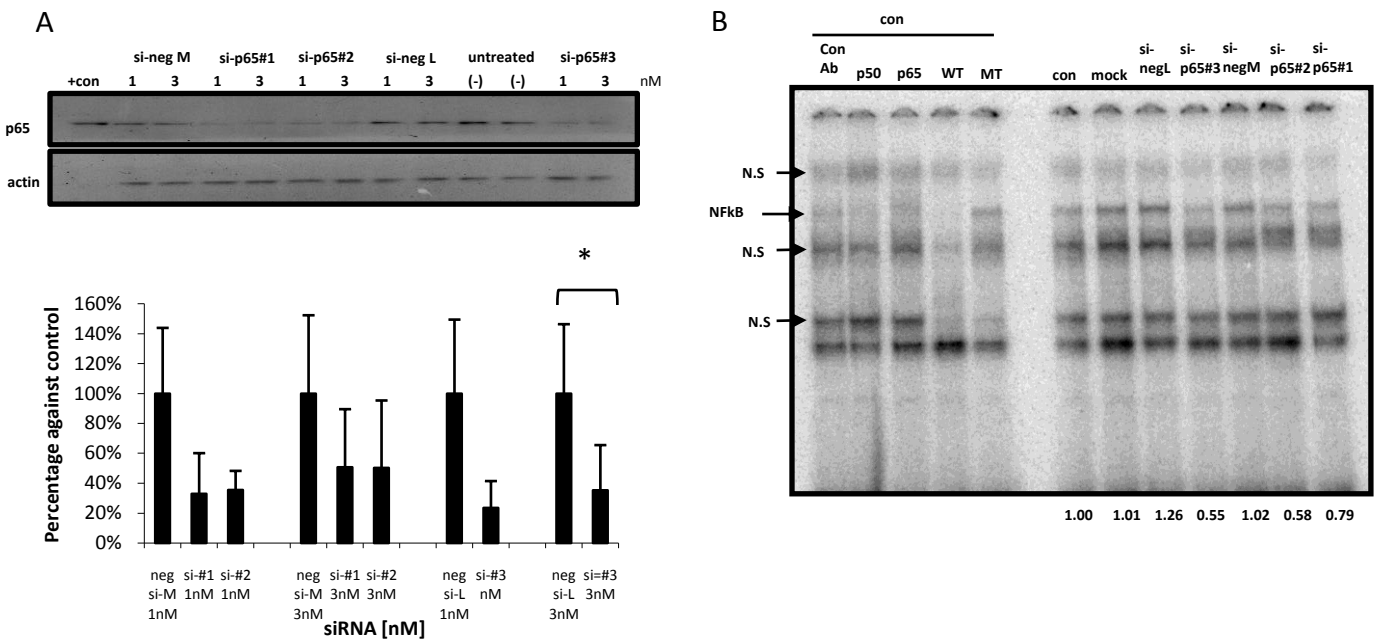

Fig 2

Supplement: Supplementary file 1 [file mmc1.pdf]
